# Combined Application of Bacterial Predation and Violacein to Kill Polymicrobial Pathogenic Communities

**DOI:** 10.1038/s41598-017-14567-7

**Published:** 2017-10-31

**Authors:** Hansol Im, Seong Yeol Choi, Sangmo Son, Robert J. Mitchell

**Affiliations:** Division of Biological Sciences, School of Life Sciences Ulsan National Institute of Science and Technology (UNIST), Ulsan, 44919 South Korea

## Abstract

Violacein is a bisindole antibiotic that is effective against Gram-positive bacteria while the bacterial predator, *Bdellovibrio bacteriovorus* HD100, predates on Gram-negative strains. In this study, we evaluated the use of both together against multidrug resistant pathogens. The two antibacterial agents did not antagonize the activity of the other. For example, treatment of *Staphylococcus aureus* with violacein reduced its viability by more than 2,000-fold with or without *B. bacteriovorus* addition. Likewise, predation of *Acinetobacter baumannii* reduced the viability of this pathogen by more than 13,000-fold, regardless if violacein was present or not. When used individually against mixed bacterial cultures containing both Gram-positive and Gram-negative strains, violacein and *B. bacteriovorus* HD100 were effective against only their respective strains. The combined application of both violacein and *B. bacteriovorus* HD100, however, reduced the total pathogen numbers by as much as 84,500-fold. Their combined effectiveness was also demonstrated using a 4-species culture containing *S. aureus*, *A. baumannii*, *Bacillus cereus* and *Klebsiella pneumoniae*. When used alone, violacein and bacterial predation reduced the total population by only 19% and 68%, respectively. In conjunction with each other, the pathogen viability was reduced by 2,965-fold (99.98%), illustrating the prospective use of these two antimicrobials together against mixed species populations.

## Introduction

Within nature, bacteria are found primarily within polymicrobial communities. This is also true of infections, which may include both pathogens and commensal microbes^1,2^. One benefit that bacteria within these communities gain is an increased resistance to antibiotics^3,4^. Two reasons for this include the presence of resistant strains^5,6^, which can degrade the antibiotic, or because the other strains act as a “sink” for the antibiotic, diluting its impact on the susceptible populations^7^. To overcome this, researchers have often resorted to using a combination of antibiotics^8–10^.

Antibiotic resistance on a single species level can be either intrinsic or acquired. Intrinsic resistance is a characteristic that is coded for in the genome of the strain, independent of antibiotic pressure and not a consequence of horizontal gene transfer. An example of intrinsic resistance includes the outer membrane in Gram-negative bacteria, which modulates the cell permeability and limits the entry of hydrophobic antibiotics^11^. Lacking an outer membrane, Gram-positive strains are generally less protected and antibiotics may enter these bacteria more easily^12^. The result is that Gram-negative bacteria are less susceptible to many antibiotics that are effective against Gram-positive pathogens.

A case in point is the bisindole antibiotic violacein, which is produced by a number of bacterial species^13^, including strains of *Chromobacterium* ^14,15^, *Janthinobacterium* ^16^, *Collimonas* ^17^ and *Duganella* ^18^. As an antibiotic, violacein is primarily active against Gram-positive strains^15,18–20^, particularly *Staphylococcus aureus* ^20–22^. It was also active against multidrug-resistant *S. aureus* ^23^, including one strain that is resistant to seven different antibiotics, *i.e*., rifampin, ciprofloxacin, clindamycin, oxacillin, erythromycin, gentamycin and tobramycin^18^. Violacein, however, is generally ineffective against Gram-negative strains.

In addition to conventional chemical-based antibiotics, several groups are also considering the use of predatory bacterial strains as living antibacterial agents^24–26^. These strains are commonly referred to as *Bdellovibrio*-and-like-organisms (BALOs). The best characterized BALO is probably *Bdellovibrio bacteriovorus* HD100, an obligate predator of bacteria that is known to attack more than 100 different human pathogens^27–30^, including multidrug resistant strains of *Acinetobacter baumannii* and *Klebsiella pneumoniae* ^28^. When it attacks its prey, *B. bacteriovorus* enters the periplasmic space and consumes its prey from within, where this predator grows, septates and eventually lyses the outer membrane of the prey to facilitate its release. In contrast with violacein, the activity of BALOs is limited to only Gram-negative bacterial strains^29–32^.

Given the inherent limitations associated with both violacein and *B. bacteriovorus*, we proposed they might be used in conjunction with one another to expand their individual spectrums of killing. To test this, *B. bacteriovorus* and violacein were partnered together and their activities evaluated using individual bacterial and polymicrobial populations, with an emphasis on those that included both Gram-negative and Gram-positive pathogens.

## Materials and Methods

### Bacterial strains and culturing techniques

The bacterial strains used in this study are listed in Table 1. Each of these strains was grown in Lysogeny Broth (LB) broth (Difco BD, USA) overnight at 30 °C and 250 rpm. To examine the impact of the treatment by predatory bacteria or violacein, the bacterial culture were prepared by centrifuging with 2,200 × g for 10 min (5430 R, Eppendorf, USA) and resuspended in HEPES buffer (pH 7.2) to an optical density (OD) of 1.0 at 600 nm wavelength. *B. bacteriovorus* HD100 was cultured as described previously^33,34^.

**Table 1:** Bacterial strains used in this study.

Bacterial Strains	Characteristics	Ref
*S. aureus* ATCC 25923		
*B. cereus* ATCC 14579		
*S. epidermidis* ATCC 14990		
*A. baumannii*	Clinical isolate	PNUH^a^
*E. coli* MG1655	Common lab strain; multidrug resistant	
*K. pneumoniae*	Clinical isolate; multidrug resistant	PNUH^a^
*S. aureus* CCARM 3090	Clinical isolate; multidrug resistant	(18)^b^

^a^Clinical strains isolated at the Pusan National University Hospital, Yangsan Campus. ^b^Obtained from the Culture Collection of Antimicrobial Resistant Microbes (http://knrrb.knrrc.or.kr/index.jsp?rrb=ccarm).

### Violacein extraction from *Pseudoduganella violaceinigra* sp. NI28 cultures

Violacein was extracted from cultures of *Pseudoduganella violaceinigra* sp. NI28 as described previously^18^. After dissolving the violacein in ethanol, the solution was filtered using a 0.22um syringe filter (Millipore, USA) and the ethanol was evaporated (EYELA N1110, Tokyo Rikakikai Co., Japan). The violacein was crystallized using an acetone wash designed to remove any contaminating salts^35^ and dissolved in DMSO (99.7% HPLC Grade, Sigma Aldrich, USA) to a final concentration of 2 g/L.

### Antimicrobial treatment

Overnight cultures of the bacterial strains were pelleted (2,200 × g, 10 min) and resuspended in HEPES buffer to an OD of 1.0 (600 nm). Each individual culture was then challenged with predatory bacteria or violacein. For the predatory tests, overnight cultures of *B. bacteriovorus* HD100 were filtered and added to the mixed bacterial suspension to achieve a predator-to-prey ratio (PPR) of 0.02~0.05. Similarly, for the violacein tests, violacein was added to the mixed bacterial suspension to a final concentration of 20 mg/ml. The cell viability (colony forming units (CFU)) was measured after each treatment. For the dual treatment experiments using predatory bacteria and violacein together, the same conditions were applied.

Likewise, for the mixed species tests, the cultures were prepared by adding equal volumes of each bacterial resuspension prior to addition of violacein and/or *B. bacteriovorus* HD100. Differences in the colony morphologies were used to differentiate between the two strains when we determined the viabilities of these cultures. For the four strain experiments, individual strains could not be differentiated between based upon the colony morphology and, so, the total viability was determined by plating serial dilutions of the cultures on LB agar plates and enumerating the colonies after 24 h at 37 °C.

### Microscopic imaging

Microscopic images were obtained using a Zeiss LSM 780 NLO microscope. To visualize the bacterial strains, dyes were used. Prior to treatment with either violacein or *B. bacteriovorus* HD100, each of the bacterial cultures were mixed with 6 µM Syto-9 (Invitrogen, USA). This is a live stain and all viable bacterial cells were fluorescently green afterwards. After washing the cells with HEPES to remove any extra dye, they were exposed to either the predatory cells (PPR of 0.1) or 20 mg/ml of violacein in HEPES. After one hour, propidium iodide (Invitrogen, USA) was added to the bacterial cultures to a final concentration of 30 µM. This dye is a dead stain and labels any non-viable bacterial cells red. After 30 minutes at room temperature, the cells were pelleted (16,000 × g, 5 min), washed and resuspended in HEPES before being imaged.

### Antibiotic resistance determination

To determine the antibiotic resistant nature of the bacterial strains, we used the same protocol as described previously^18^. Strain resistance was determined using the latest breakpoint tables available at the European Committee on Antimicrobial Susceptibility Testing (EUCAST) website (http://www.eucast.org/clinical_breakpoints/).

### Spot viability assay

To compare the activity of conventional antibiotics and the dual treatment protocol used in this study, we tested representative bacteriostatic and bactericidal antibiotics, *i.e*., chloramphenicol and gentamycin, respectively. For the assays, a concentrated dosage that was 10-fold higher than the typical dose used in labs was employed. As such, the bacterial community was exposed to either 350 mg/l chloramphenicol, 500 mg/l gentamicin or both. After treatment, the cells viabilities were evaluated using a spot viability plate. For this, the exposed cultures were serially diluted into LB media and 10 µl was spotted on LB agar within a square petridish (SPL, Korea) and incubated for 24 hour at 37 °C. The extent of growth was used as an indication of viability.

### Statistical analysis

In this research, all assays were accomplished at least three replicates and the standard deviations among the samples are indicated with error bars on the graphs. Statistical analysis was done using Student’s t-test to compare two sets of results and the statistical significance was marked on the graphs using the marks *, **, *** for *p-*values of less than 0.05, 0.01, and 0.001 respectively. For comparing three or more data sets, analysis of variance (ANOVA) tests were performed followed by the Tukey post-hoc test. Statistically, significantly different groups using a *p*-value < 0.05 are shown on the graphs using letters (a, b, c and d).

## Results

### Activity Spectrum of Violacein and Bacterial Predation

**Figure 1:**
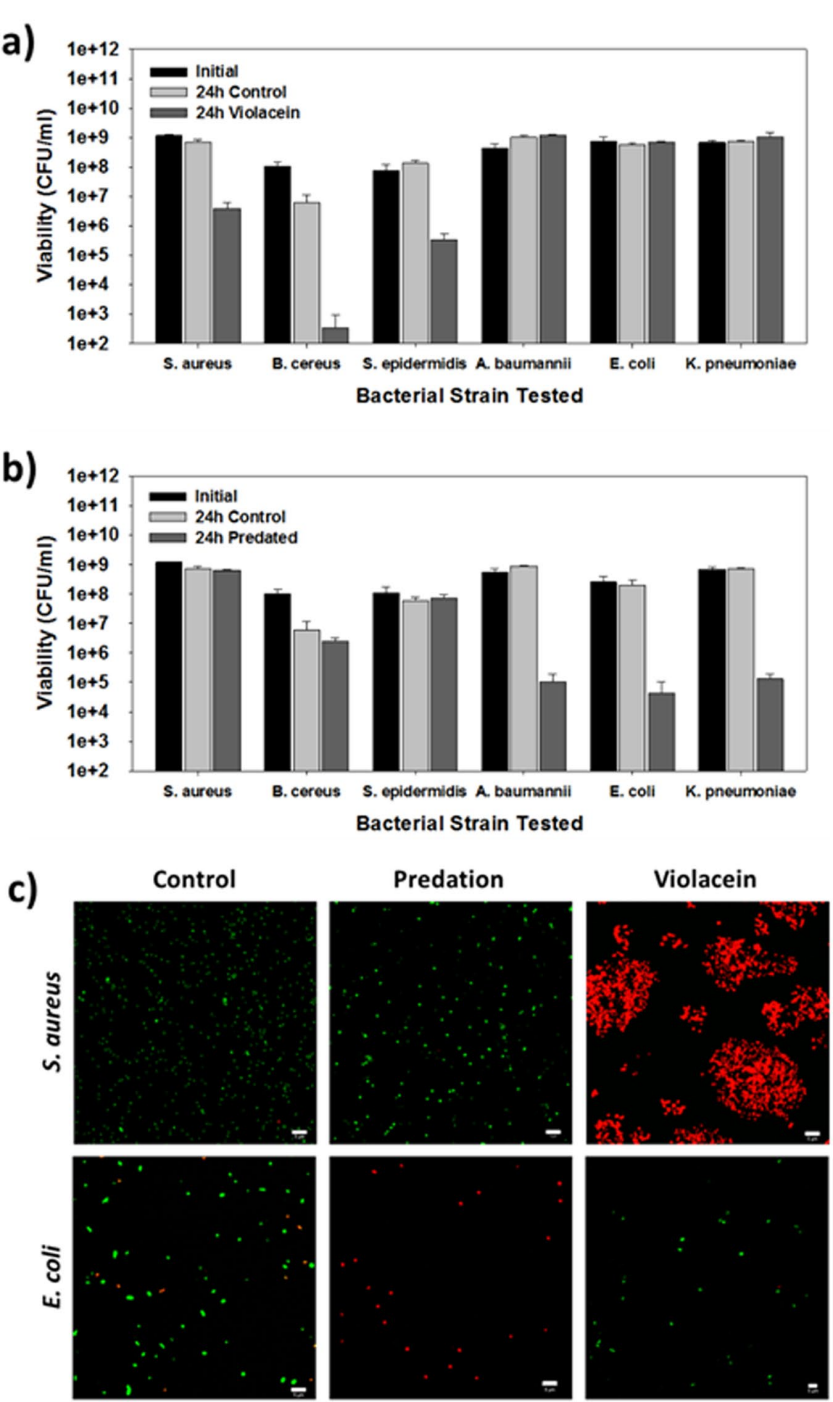
Spectrum of activity for violacein and *B. bacteriovorus* HD100. (**a** and **b**) The activity of each antimicrobial was evaluated using three Gram-positive and three Gram-negative bacterial strains. The pathogens were treated with either (**a**) 20 mg/l violacein or (**b**) 1 × 10^7^ PFU/ml of *B. bacteriovorus* HD100. The viabilities of the pathogens were determined initially and after 24 hrs (n = 4). (**c**) Impact of violacein and *B. bacteriovorus* HD100 on *S. aureus* and *E. coli*. The bacteria were treated with either 20 mg/l violacein or approximately 1 × 10^7^ PFU/ml of *B. bacteriovorus* HD100 for 1 hr. Using the Live/Dead BacLight kit, the viable and non-viable bacteria were fluorescently labeled and imaged using confocal microscopy. Scale bar – 10 µm.

Violacein is quite active against *S. aureus*, killing more than 99% of the culture when added at a concentration of 20 mg/L or greater^18,21,23^. Using this concentration, we tested the activity of violacein against six different strains (Tables 1 and 2), including three Gram-positive and three Gram-negative species. As shown in Fig. 1a, only the Gram-positive cultures were sensitive to violacein, with each showing significant viability losses after 24 hours. In contrast, none of the Gram-negative cultures were negatively impacted by violacein (Fig. 1a). The *A. baumannii* and *K. pneumoniae* strains used throughout this study were multidrug resistant clinical isolates (Table 2).

**Table 2:** Antibiotic resistance of the clinical isolates used in this study. The highlighted values are indicative of resistance.

	AMP	CIP	GEN	TET	CM	RIF	ERY	CC
*A. baumannii*	0(R)	0(R)	0(R)	18–22(S)	12–15(I)	15–17(S)	15–17(S)	ND
*K. pneumoniae*	9–10(R)	29–30(S)	20–21(S)	24–25(S)	23–25(S)	6–8(R)	6–8(R)	ND
*S. aureus* CCARM 3090	9–10(R)	0(R)	23–25(S)	9–10(R)	22–25(S)	30–35(S)	0(R)	0(R)

AMP: ampicillin, CIP: Ciprofloxacin, GEN: Gentamycin, TET: Tetracycline, CM: Chloramphenicol, RIF: Rifampicin, ERY: Erythromycin, CC: Clindamycin. Classifications: (R) – Resistant; (I) – Intermediate; (S) – Susceptible; ND – Not determined.

The results with *B. bacteriovorus* HD100 stood in stark contrast as only the Gram-negative strains experienced a loss in their viabilities (Fig. 1b). These results clearly illustrate the different activity spectrums for both antimicrobials and highlight the limitations of each. This dichotomy is further demonstrated in Fig. 1c where *S. aureus* or *E. coli* were imaged after being exposed to either. In agreement with Fig. 1a and b, violacein only killed *S. aureus* while *B. bacteriovorus* HD100 only killed *E. coli*.

### Violacein and *B. bacteriovorus* Do Not Adversely Impact Each Other’s Activity

**Figure 2:**
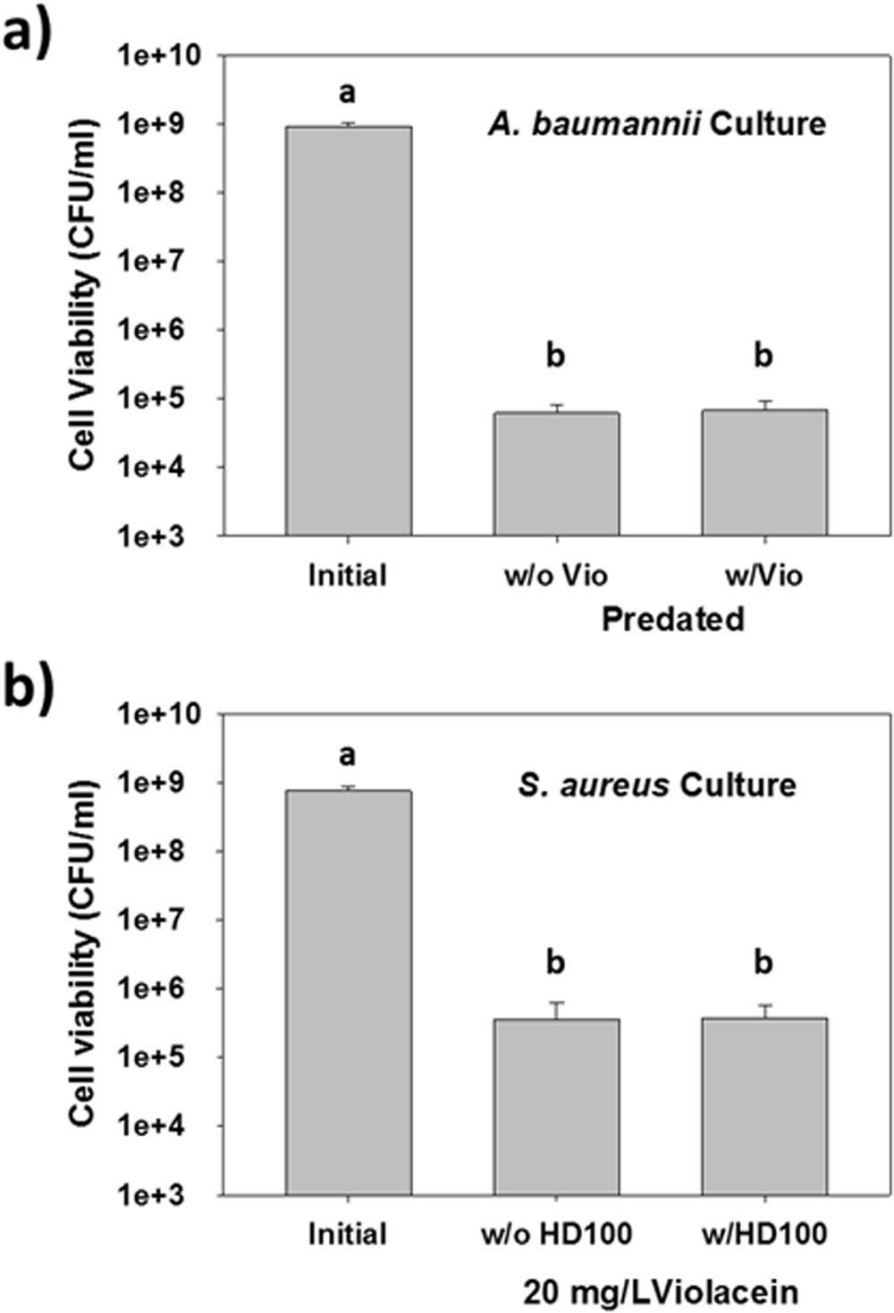
Violacein and *B. bacteriovorus* HD100 do not hinder the activity of the other. (**a**) Predation of *A. baumannii* with or without addition of 20 mg/l violacein. *A. baumannii* was predated on by *B. bacteriovorus* HD100 as effectively when violacein was present as when it was omitted. The viabilities were measured after 24 h, as in Fig. 1. a and b = *p* < 0.05 (n = 3). **(b**) The activity of violacein against *S. aureus* with or without addition of *B*. *bacteriovorus*. The presence of the predatory bacterium did not negatively impact the activity of violacein against this pathogen. The viabilities were measured after 24 h, as in Fig. 1. a and b = *p* < 0.05 (n = 3).

The above data shows violacein was not active against the three Gram-negative strains. Given *B. bacteriovorus* HD100 is also Gram-negative, we speculated that violacein would also not be harmful towards this predator or its activity. Figure 2a shows that this was the case when *A. baumannii* was used as the prey strain. In both cases, the *A. baumannii* populations were similarly reduced by more than 4-log due to the activity of *B. bacteriovorus* HD100. Similarly, the activity of violacein was not thwarted by the presence of *B. bacteriovorus* HD100, as illustrated in Fig. 2b. This figure shows reduction of the *S. aureus* populations was comparable whether or not the predator was added.

### The Activities of Violacein and *B. bacteriovorus* are Compatible with Each Other

We next tested the possibility of using both treatments together against cultures containing both a Gram-positive and a Gram-negative strain. For these tests, we selected *S. aureus* (average initial population: 4.2 × 10^8^ CFU/ml) and either *A. baumannii* or *K. pneumoniae* (average initial population: 7.9 × 10^8^ CFU/ml).

**Figure 3:**
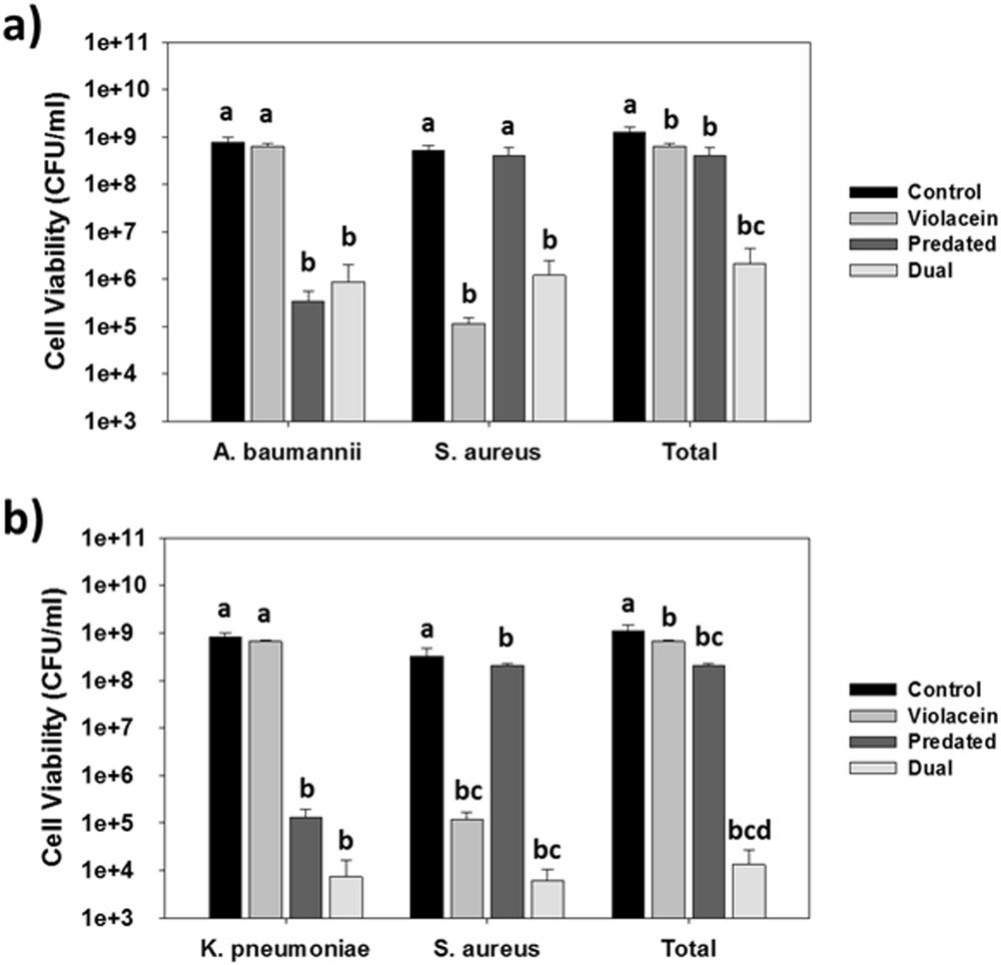
Treatment of two-strain cultures containing *S. aureus* with violacein and *B. bacteriovorus* HD100. (**a**) Impact of 20 mg/l violacein alone, 1 × 107 PFU/ml *B. bacteriovorus* HD100 alone or both together against a mixed culture containing *S. aureus* and *A. baumannii*. The viabilities were measured after 24 hr. Each of the antimicrobials was effective against their respective pathogen when used alone, but together led to a significant loss in the total pathogen numbers. a, b and c = *p* < 0.05 (n = 3). (**b**) Impact of 20 mg/l violacein alone, 1 × 107 PFU/ml *B. bacteriovorus* HD100 alone or both together against a mixed culture containing *S. aureus* and *K. pneumoniae*. The viabilities were measured after 24 hr. Each of the antimicrobials was effective against their respective pathogen when used alone, but together led to a significant loss in the total pathogen numbers. a, b, c and d = *p* < 0.05 (n = 3).

As shown in Fig. 3a, both antimicrobials were active against their respective pathogen: treatment with violacein alone killed only *S. aureus* while the use of *B. bacteriovorus* HD100 by itself only reduced the *A. baumannii* population. As a result, the total pathogen number did not decrease very much, *i.e*., only 51% with violacein and 69% with *B. bacteriovorus* HD100. When violacein and *B. bacteriovorus* HD100 were used together, however, both pathogens were significantly killed and the total pathogen numbers were reduced by 99.8% (620-fold). Similar results were obtained when *S. aureus* and *K. pneumoniae* were cultured together (Fig. 3b) as individual treatments with violacein and *B. bacteriovorus* HD100 led to a 41% and 81% loss in total pathogens, respectively, while a dual treatment caused a 99.999% (84,500-fold) reduction in the pathogen viability.

**Figure 4:**
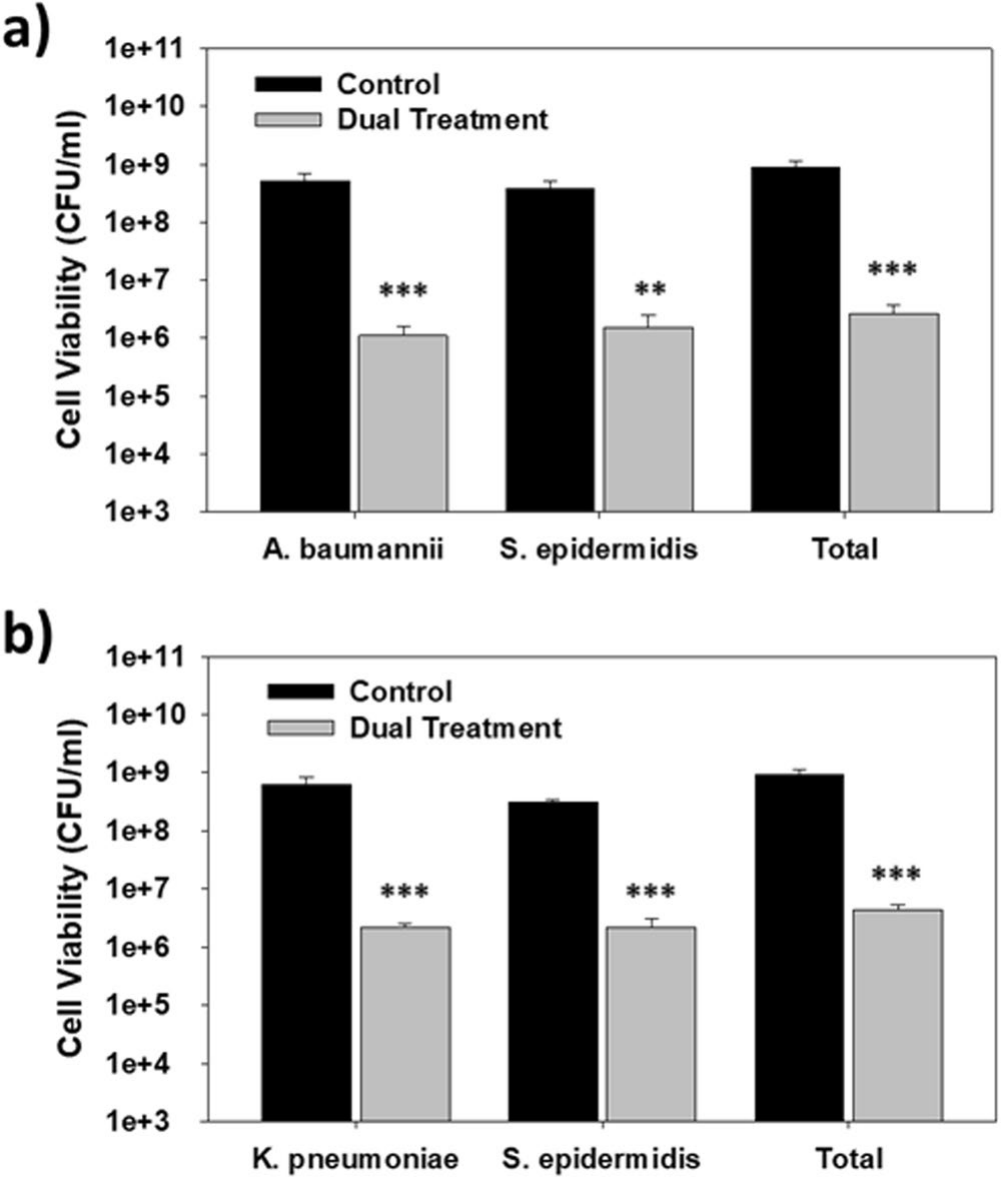
Treatment of two-strain cultures containing *S. epidermidis* with violacein and *B*. *bacteriovorus* HD100. (**a**) Dual treatment of a mixed culture containing *S. epidermidis* and *A*. *baumannii* using 20 mg/l violacein and 1 × 10^7^ PFU/ml *B. bacteriovorus* HD100. The viabilities were measured after 24 hr (**p < 0.001; ***p < 0.001) (n = 3). (**b**) Dual treatment of a mixed culture containing *S. epidermidis* and *K. pneumoniae* using 20 mg/l violacein and 1 × 10^7^ PFU/ml *B. bacteriovorus* HD100. The viabilities were measured after 24 hr (***p < 0.001) (n = 3).

Dual treatment tests were also performed using either *A. baumannii* or *K. pneumoniae* alongside a different Gram-positive bacteria, *i.e*., *S. epidermidis* (Fig. 4). In both cultures, the viability of each pathogen was reduced by more than 99.3%.

### Application of the Dual Treatment to a Complex Microbial Community

**Figure 5:**
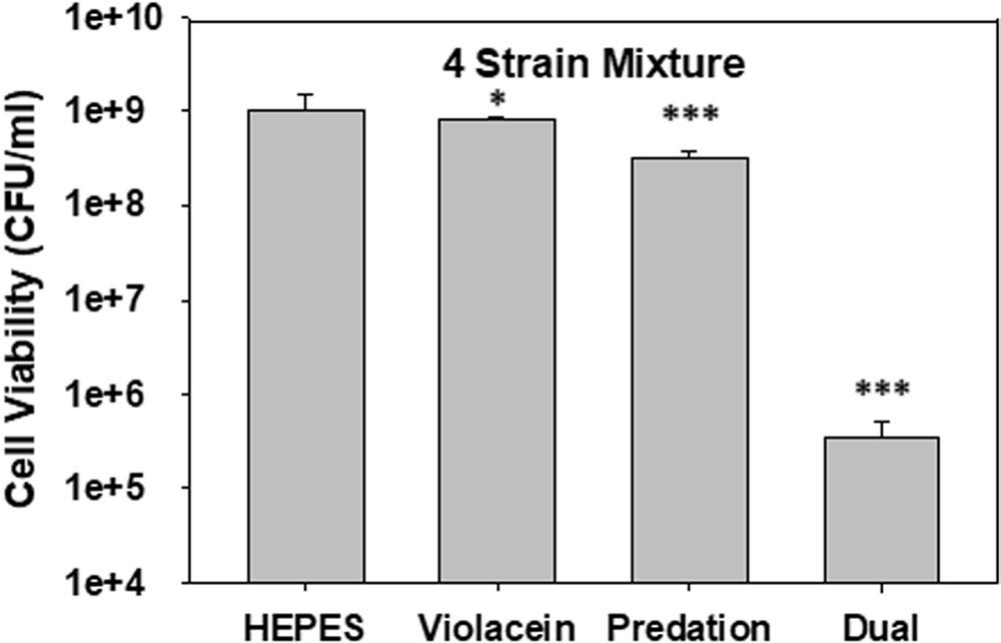
Dual treatment of polymicrobial culture containing four different pathogens. Impact of 20 mg/l violacein alone, 1 × 10^7^ PFU/ml *B. bacteriovorus* HD100 alone or both antimicrobials together against a culture containing four pathogens (*S. aureus*, *B. cereus*, *A. baumannii* and *K. pneumoniae*, Table 3). The viabilities of the culture as a whole were measured after 24 hr. Each of the antimicrobials led to mild but significant losses when used alone, but together led to a 99.96% loss in the total pathogen numbers (*p < 0.05; ***p < 0.001) (n = 3).

Subsequently, we tested if a dual treatment is also effective against a complex microbial consortium harboring four different human pathogens, *i.e*., *S. aureus*, *A. baumannii*, *B. cereus* and *K. pneumoniae* (Fig. 5). The population of each pathogenic strain is listed in Table 3, with a total initial population of 5.9 × 10^8^ CFU/ml. After treatment with either *B. bacteriovorus* HD100 or violacein alone, the Gram-negative and Gram-positive strain viabilities decreased, respectively, but we could not reliably measure the impact due to the overwhelming presence of the surviving strains. The use of violacein alone resulted in a 19% reduction of the total number of pathogens while *B. bacteriovorus* HD100 led to a 68% loss (3.1-fold). As shown in Fig. 5, when both antimicrobials were used, the number pathogens was reduced by 99.96% (2,970-fold).

**Table 3:** Impact of predation or violacein on the survival of the four pathogens within a mixed population.

	Initial	HEPES (24 hr)	Violacein (24 hr)	Predation (24 hr)
*B. cereus*	8.9 ± 1.4 × 10^6^	1.8 ± 0.7 × 10^7^	N.D.^a^	3.6 ± 1.3 × 10^7^
*S. aureus ATCC 25923*	2.7 ± 0.4 × 10^8^	3.1 ± 0.2 × 10^8^	N.D.	2.9 ± 0.7 × 10^8^
*K. pneumoniae*	9.7 ± 0.7 × 10^7^	3.5 ± 0.2 × 10^8^	4.2 ± 0.1 × 10^8^	N.D.
*A. baumannii*	2.1 ± 0.5 × 10^8^	3.5 ± 0.8 × 10^8^	4.2 ± 0.3 × 10^8^	N.D.
Total (Average)	5.9 × 10^8^	1.03 × 10^9^	8.4 × 10^8^ (81%)	3.2 × 10^8^ (32%)

^a^N.D. – Not detected on the agar plates with a 10^7^ dilution.

### Dual Treatment Impacts on Multidrug Resistant Bacterial Populations

**Figure 6:**
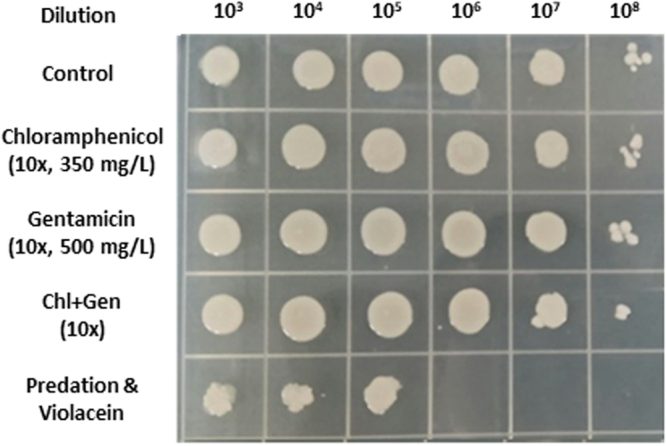
Dual treatment of a culture containing multidrug resistant strains of *A. baumannii* and *S. aureus*. A mixed culture containing *A. baumannii* alongside *S. aureus* CCARM 3090 was treated with either chloramphenicol, gentamicin, these two antibiotics together (Chl + Gen) or the dual treatment (Predation & Violacein). The concentrations of the two antibiotics were 10x higher than commonly used in lab experiments. After 24 hr, the cultures were serially diluted and 10 µl spotted onto an agar plate. The image here shows the resulting growth, illustrating the much higher degree of killing obtained with the dual treatment protocol.

As a final test using both predatory bacteria and violacein together, a test culture containing multidrug resistant *S. aureus* (Table 2) was prepared. The other strain was the same *A. baumannii* as above, which is also multidrug resistant. Both of these strains are resistant to numerous antibiotics, as listed in Table 2, and the initial population of each pathogen within the cultures was 5.2 × 10^8^ and 3.2 × 10^8^ CFU/ml, respectively. For comparison, we also treated the mixed pathogen culture with a concentrated blend of gentamicin with chloramphenicol (Fig. 6). The image show the use of *B. bacteriovorus* HD100 and violacein was much more effective at killing the pathogens than the use of gentamicin and chloramphenicol, even though the concentrations of the two antibiotics were much higher than what is typically used.

## Discussion

Due to the universal spread of antibiotic resistance, it is projected that the number of human mortalities each year resulting from antimicrobial resistant pathogens will surpass that caused by cancer by 2050^36^. Two alternative antimicrobials currently being studied by various groups are violacein and predatory bacteria^18,23,25,28,37,38^. In addition to their antibiotic activities, both also show promising toxicology results, a key characteristic for broad spectrum antibiotics^8,39^; in tests with mice, violacein was non-toxic at concentrations as high as 1 mg/kg^40^ while *B. bacteriovorus* was not toxic towards several different animal hosts^41–43^ or in human cell cultures^44–46^.

Treatment of *S. aureus* with violacein not only led to a significant loss of viability but also caused a substantial degree of aggregation. Similar results were seen in other studies where *S. aureus* was exposed to either violacein^22^ or galangin^47^. In the former study, violacein was shown to disrupt the membrane integrity of *S. aureus*, which led to a rupturing of the cell membrane and leakage of the cellular components. It was hypothesized by Cushnie *et al*. (2007) that galangin similarly damages the membrane and exposes the hydrophobic regions located within the phospholipid bilayer, which then interact and cause aggregation^47^. The same process likely occurs with violacein, which would account for the cell aggregates seen here. The activity of violacein also helps to explain the inherent resistance of Gram-negative bacterial strains since the outer membrane would act like a sponge and absorb violacein, protecting the inner membrane. With *B. bacteriovorus*, however, only *E. coli* was killed while the *S. aureus* cells remained viable (Fig. 1c). This is a clear demonstration that *S. aureus* is not a prey for *B. bacteriovorus* and reaffirms the long held knowledge that BALOs only predate and consume Gram-negative strains^29–32^, despite claims in one study that *S. aureus* is also attacked^48^. Although the mode of activity for violacein has recently been resolved, much still remains unanswered about how predatory bacteria recognize, enter and kill their prey and the processes involved in each of these steps.

The results above illustrate inherent limitations for both violacein and *B. bacteriovorus* HD100; violacein is largely effective against Gram-positive bacteria while *B. bacteriovorus* HD100 attacks only Gram-negative strains. Given the different activity spectrums for these two antimicrobials, we proposed they might be complementary and used alongside each other against polymicrobial populations. However, it was not known if predatory bacteria and violacein negatively impact the activity of the other. Here we show that this was not the case – violacein was just as effective against *S. aureus* regardless of whether *B. bacteriovorus* was present while predation of *A. baumannii* proceeded just as successfully in the presence or absence of violacein. This suggested that they can be used together and hinted at their potential use to eradicate polymicrobial populations.

Polymicrobial infections are a serious problem since complex bacterial communities can benefit from the properties of each of the members. An example of this is the protection afforded to a susceptible population by a resistant member of the community, such as through enzymatic inactivation of the antibiotic^5,6^. Another possibility is the “inoculum effect”, whereby the antibiotic activity is diluted out by the presence of other bacteria, which act as “sinks” for the antibiotic and limit its effectiveness against susceptible microbe populations^7^. We were concerned that this may be a problem for violacein. Violacein is a hydrophobic compound and attacks cellular membranes, implying the presence of naturally-resistant Gram-negative bacterial cells may act as a “sink” for this antibiotic and dilute its impact on *S. aureus* or other Gram-positive pathogens. The results, however, suggest that this is not a problem as violacein at the concentration used was as effective at killing *S. aureus* regardless if *A. baumannii* or *K. pneumoniae* were present.

Likewise, although they are not attacked by *B. bacteriovorus*, Gram-positive strains may act as decoys and slow the predation of susceptible Gram-negative bacterial strains^49^. As *B. bacteriovorus* does not display a clear chemotaxis towards prey cells^50^, it is generally thought that interactions between *B. bacteriovorus* HD100 and its prey are random events that occur as the predator swims within the media. By extrapolation, this means *B. bacteriovorus* will also come in contact and interact with non-prey bacterial cells. Such interactions have been observed with both *Neisseria gonorrhoeae* ^51^ and *Bacillus subtilis* ^49^, although neither is predated upon. Stemming from this reasoning, Wilkinson (2001) developed the first mathematical model to describe the impact decoys may have on *B. bacteriovorus* predation rates and efficacies using a double-Monod framework based upon a continuous flow system^52^. Hobley *et al*. (2006) followed this with experimental data using *B. subtilis* as a decoy in predation cultures with *E. coli* S17-1 as the prey, and developed their own model based upon the Lotka-Volterra equations^49^. They found predation rates were slower during the first seven hours when *B. subtilis* was present as a decoy at a ratio of approximately 1:2 (decoy:prey). In contrast, the study by Van Essche *et al*. (2010) found the presence of a decoy, *i.e*., Gram-positive *Actinomyces naeslundii* ATCC 12104, at a 14-fold excess had no impact on predation of *Aggregatibacter actinomycetemcomitans* ATCC 43718^53^. Similar results were reported by Loozen *et al*. (2014) in their study with a six-member bacterial community^54^. The six species present within the community were all oral bacteria and included four Gram-negative and two Gram-positive species. Along with the Gram-positive bacterial strains, two of the Gram-negative strains, *i.e*., *Porphyromonas gingivalis* and *Prevotella intermedia*, were not predated upon by *B. bacteriovorus* HD100. However, *A. actinomycetemcomitans* and *Fusobacterium nucleatum* are both prey and the presence of the four other strains did not hinder their predation.

Clearly some non-prey bacteria can act as decoys and slow down predation initially^49^, while others have no impact^53,54^. Here, we found the presence of *S. aureus* did not negatively impact predation. Similar results were seen with *S. epidermidis*. One clear difference between *S. aureus* and *S. epidermidis*, though, was the viabilities after a dual treatment with *K. pneumoniae*. With *S. epidermidis*, the viabilities were comparable with those seen after single treatments in Fig. 1. With *S. aureus*, however, the viabilities of both pathogens were reduced further by the dual treatment (Fig. 3).

The activities of violacein and *B. bacteriovorus* HD100 were also evaluated using a four-member pathogen culture, with positive results. As with the two-strain cultures, the presence of the four different strains did not significantly deter the activities of either antimicrobial when used alone (Table 3) nor when used together (Fig. 5). In the latter case, the number of viable pathogens was reduced by more than 3-log and stands as a clear demonstration the potential these two antimicrobials have when used together.

Not only are violacein and *B. bacteriovorus* HD100 effective against their respective classes of bacteria, they are also both active against multidrug resistant pathogens. For violacein, the minimum inhibitory concentrations for a wild-type *S. aureus* and four other strains, including a clinical isolate that is resistant to seven different antibiotics, were identical^18^. Along the same lines, the antibiotic resistant nature of the prey had no obvious impact on the ability of *B. bacteriovorus* to attack^28^, while predation reduces the presence of the antibiotic resistance marker by degrading the prey DNA^55^. Consequently, violacein and *B. bacteriovorus* are both active against multidrug resistant pathogens. This was illustrated here in experiments with a mixed culture containing multidrug resistant strains of *S. aureus* and *A. baumannii*. For comparison, we also exposed this culture in parallel to high doses of chloramphenicol, gentamicin or both of these antibiotics. With chloramphenicol and/or gentamicin, the culture viabilities were not significantly impacted, even though the concentration of each antibiotic was 10-fold higher than commonly employed in the lab. In contrast, a dual treatment with violacein and *B. bacteriovorus* HD100 led to a 2- to 3-log loss, a result that is indistinguishable with that in Fig. 3 with the wild-type *S. aureus*.

In conclusion, this study evaluated the activities of violacein and *B. bacteriovorus* HD100 when used together against polymicrobial cultures. Violacein, being active primarily against Gram-positive strains such as *S. aureus*, and *B. bacteriovorus* HD100, which attacks only Gram-negative bacteria, were found to be compatible and not diminish the activity of the other. This was demonstrated in cultures containing two and four different bacterial species, including both Gram-positive and Gram-negative strains. When used alone, the overall viabilities decreased marginally (less than 1-log reduction) but, when violacein and *B. bacteriovorus* HD100 were used together the number of viable pathogens was reduced by 3-log or greater, even when multidrug resistant pathogens were present.
